# Identifying Platelet Lipidomic Networks and Evaluating Machine-Learning Models to Identify Distinctive Features Between Chronic and Acute Coronary Syndrome

**DOI:** 10.3390/cells15131190

**Published:** 2026-06-30

**Authors:** Vivek Nandhan Kanpa, Suzy Whoriskey, Ana Le Chevillier, Jean de Villiers, Adrian Brun, Tobias Harm, Manuel Sigle, Kristina Dittrich, Andreas Goldschmied, Dominik Rath, Michael Lämmerhofer, Patricia B. Maguire, Meinrad P. Gawaz, Luisa Weiss

**Affiliations:** 1 UCD Conway SPHERE Research Group, Conway Institute, University College Dublin, D04 V1W8 Dublin, Ireland; vivek.kanpa@icahn.mssm.edu (V.N.K.);; 2 AI Healthcare Hub, Institute for Discovery, O’Brien Centre for Science, University College Dublin, D04 V1W8 Dublin, Ireland; 3 Windreich Department of AI & Human Health, Icahn School of Medicine at Mount Sinai, New York, NY 10029, USA; 4 School of Mathematics and Statistics, University of Strathclyde, Glasgow G12 8QQ, UK; 5 School of Biomolecular and Biomedical Science, University College Dublin, D04 V1W8 Dublin, Ireland; 6 SAS Institute Ireland, 2 Windmill Lane, Dublin Docklands, D02 K156 Dublin, Ireland; 7 Institute of Pharmaceutical Sciences, Eberhard Karls University Tübingen, 72076 Tübingen, Germany; 8 Department of Cardiology and Angiology, University Hospital Tübingen, Eberhard Karls University Tübingen, 72074 Tübingen, Germany

**Keywords:** multi-omics, platelets, coronary disease, machine learning

## Abstract

Platelet lipidomics offers a window into the thromboinflammatory responses to acute coronary syndromes (ACS) and chronic coronary syndromes (CCS), yet differences between the platelet lipidomes of these two coronary artery disease subtypes remain poorly characterized. In this pilot study, untargeted platelet lipidomics and miRNA transcriptomics were performed on platelets isolated from 19 ACS and 57 CCS patients undergoing coronary angiography, integrated with routine clinical and hematological traits in statistical network analyses and cross-validated machine learning models. Platelet lipidomics identified 81 lipid features significantly altered between ACS and CCS (FDR *q* < 0.05), predominantly involving phosphatidylcholines, lysophosphatidylcholines, and ceramides. Cer 18:2;O2/24:0 showed the strongest inverse association with ACS of any identifiable lipid (*q* = 0.0394, OR = 0.297, 95% CI [0.171, 0.515]), while increased Cer 18:0;O2/22:1 (*q* = 0.088, OR = 3.072, 95% CI [1.607, 5.878]) and Cer 18:0;O2/18:0 (*q* = 0.088, OR = 2.791, 95% CI [1.515, 5.145]) demonstrate nominally significant promotive effects on ACS. Differential correlation analysis further identified oxidized phospholipid species as connectivity hubs in ACS-specific lipid networks, a pattern not detectable by differential abundance approaches alone. Although untargeted lipidomics did not meaningfully outperform a routine clinical trait benchmark in ACS classification, phosphatidylcholine-trained models achieved modest gains in AUROC, and the prominent ceramide signal—platelet-specific and mechanistically distinct from plasma ceramide risk scores—supports a targeted validation strategy centered on a ceramide-enriched platelet lipid panel in prospective, adequately powered cohorts.

## 1. Introduction

Acute coronary syndromes (ACS) are one of the major causes of death worldwide, and a main concern in patients presenting to the emergency department with acute chest pain [1]. Rapid and reliable diagnosis is vital for appropriate treatment and correlates with survival [2]. Electrocardiography (ECG) can rapidly identify a subset of patients with ST segment elevation; however, the majority of patients do not present such ST elevation and therefore require biomarker assessment [3]. For decades, clinicians have attempted to improve approaches to rapidly diagnose ACS subtypes. Standard statistical methods, such as linear regression-based analysis of traditional risk factors, aim to identify differences on a population level and include a limited number of variables [4,5]. Conversely, machine-learning (ML)-based approaches utilize patient-level data to identify and extract outcome predictors, ultimately generating reliable models. Recent advancements in the development of ML-based algorithms may, therefore, provide a useful platform for rapid patient stratification [6]. Indeed, incorporation of additional blood-based biomarkers using ML models could enhance diagnostic and prognostic performance compared to basic rule-based algorithms [7,8,9] by identifying latent structures in the realm of clinical patient data that may remain undetected by clinicians.

Platelets are a vital contributor to the pathophysiology of ACS and changes in platelet reactivity correlate with an increased risk of adverse cardiovascular events [4,5]. Furthermore, ACS is associated with substantial changes in the platelet proteome [10], lipidome [11,12] and transcriptome [13]; hence, addition of platelet-derived molecules may further improve risk prediction and diagnostic accuracy of ML models. While platelet proteomic and transcriptomic alterations have been studied extensively in ACS, lipidomic changes have only recently been investigated. While plasma lipidomic profiles can mirror the biochemical effects of both genetic and lifestyle influences, contribution from multiple tissues and circulating lipoproteins, [14,15,16], platelet lipidomics offers several conceptual and biological advantages, making their use as potential biomarkers especially valuable in relation to cardiovascular diseases. Given that platelets are active mediators of thrombosis and vascular inflammation, they represent a biologically relevant cellular compartment in ACS pathogenesis. Platelet lipidomics captures lipid remodeling associated with platelet activation, membrane turnover, granule secretion, extracellular vesicle release, and platelet–endothelial interactions, providing mechanistic insights that are not readily obtainable from plasma analyses alone [12,17,18]. Furthermore, intracellular lipid alterations may be detected before they become diluted within the circulating lipid pool, potentially enhancing sensitivity to disease-related changes.

In this preliminary pilot study, we employed ML techniques on a comprehensive dataset derived from a prospective cohort study of 76 patients who experienced symptomatic coronary artery disease (CAD). We, for the first time, combined untargeted platelet lipidomics with targeted cardiovascular miRNA transcriptomics and demographic and clinical data to identify lipid signatures that discriminate ACS from CCS and to characterize the biological pathways associated with these alterations. Given the crucial contribution of platelets to CAD, we aimed to explore whether platelet-derived lipidomic features contain signals capable of discriminating ACS from CCS among patients with established CAD, and to identify candidate molecular features for future validation in larger cohorts.

## 2. Methods

### 2.1. Study Population

Data were collected in a single-centre prospective cohort study enrolling 139 consecutive patients who presented with symptomatic CAD at University Hospital Tübingen, Germany. All patients were treated for CAD, and the severity of the disease was analysed by coronary angiography within 24 h of hospital admission. Of the 139 patients, lipidomic analysis was performed in 107 patients, and miRNA profiling was completed in 100 patients. To allow data integration, the 76 patients that provided both platelet lipidomic and miRNA data were selected as the final cohort analyzed in this study.

Among multi-omics assayed patients with acute coronary syndrome (ACS; *n* = 19) or chronic coronary syndrome (CCS; *n* = 57), anonymized data describing baseline anthropometric and clinical factors (age, gender, outcome, medication status, and medical history), and haematological laboratory parameters (complete blood count, biochemistry panel) were compiled. ACS was defined according to contemporary ESC guidelines, and diagnosis was based on the presence of acute myocardial ischemia determined by clinical symptoms, electrocardiographic abnormalities, dynamic changes in cardiac biomarkers indicative of myocardial injury, and coronary angiographic findings where appropriate. CCS patients were defined as individuals with stable coronary artery disease undergoing elective coronary angiography in the absence of an acute coronary event [19,20,21,22]. Inclusion criteria included symptomatic CAD (at least one hemodynamic relevant >50% lumen narrowing coronary artery stenosis). Patients without CAD were excluded from this study. The study was approved by the Ethics Committee at the Medical Faculty of the Eberhard Karls University #270/2011B01 (8 August 2011) and #237/2018BO2 (18 April 2018), and all patients gave written informed consent. The study complied with the Declaration of Helsinki and the Good Clinical Practice Guidelines.

### 2.2. Blood Sampling and Platelet Preparation

Blood samples for platelet isolation were collected from the arterial sheath at time of coronary angiography before stent placement and administration of unfractionated heparin according to a standardized protocol. Platelet isolation and mass-spectrometry sample preparation were performed as previously described [12,18,23].

### 2.3. Predictors and Outcomes

Routine clinical haematology laboratory data (*n* = 37) were combined with lipidomics data, i.e., known lipid species (structurally annotated by spectral match to LipidBlast library) (*n* = 939) and unknown features (*n* = 2973) detected by mass spectrometry, as well as miRNA array (*n* = 63) data reflected cardiovascular disease-associated miRNAs and utilized as input variables for variable selection. Patient outcome was defined as positive or negative diagnosis of acute coronary syndrome as above.

### 2.4. Data Processing

Clinical and patient anthropometric data were obtained from patients at the time of hospital admission and collated with platelet miRNA array (*n* = 63) and lipidomic (*n* = 3912) data. Lipidomic data included known lipid species (structurally annotated by spectral match to LipidBlast library) (*n* = 939) and unknown features (*n* = 2973) detected by mass spectrometry. Data were preprocessed by normalizing and standardizing of continuous measurements (complete blood counts, lipidomics, miRNA-omics), one-hot encoding categorical phenotypes, median imputing variables with less than 20% missing data, and dropping variables in excess of 20% data missingness, resulting in the exclusion of solely miRNA transcript miR34aNND. To train machine-learning models, this preprocessing pipeline was applied within each cross-fold, rather than across the complete cohort, to minimize data leakage bias. All remaining variables were utilized for the complete case analysis of the dataset.

### 2.5. Exploratory Analysis

Platelet miRNA array, lipidomic, and clinical and patient clinical data were evaluated for differential association with a binary ACS or CCS outcome within CAD via Welch’s *t*-tests for continuous traits or Fisher’s exact tests for binary traits. Univariate logistic regressions were independently fitted on each molecule from lipidomics on the complete patient dataset (*n* = 76) to measure the effect size of each molecular species on CAD binary outcomes, adjusted for age and sex covariates. Odds ratios for each molecule were derived from each model’s estimated beta coefficient. Identifiable lipid species were additionally grouped by their structural class to evaluate class-wide enrichment and associations with ACS versus CCS.

### 2.6. Differential Correlation Analysis of CAD Lipidome and Transcriptome

Within ACS (*n* = 19) and CCS (*n* = 57) cohorts, pairwise Spearman correlation coefficients were computed between lipids and transformed via Fisher’s r-to-z transformation. A Δz distance matrix was computed as the difference between z-scores in each distance matrix, and a *p*-value corresponding to each lipid pair’s Δz was calculated via the cumulative distribution function (CDF). This resulted in identifying whether, for each pair of molecules, there was a significantly different correlation observed in ACS and CCS cohorts. A rewiring network of significantly altered connections was plotted, and an exploration of differential molecular expression between ACS and CCS cohorts was conducted on named lipid molecules.

Identifiable lipid molecules with nominal (*p* < 0.05) or significant (*q* < 0.05) rewiring were selected for ranked lipid set-enrichment analysis using logistic regression-derived beta coefficients, via LION/web ontology [24]. Then, the average change in network connectivity (ACS–CCS node degree) for each lipid molecule’s ontology association was computed and compared to all other lipidomic ontology associations to determine whether each ontology class is more rewired toward ACS (versus CCS) given the overall network rewiring patterns observed. Because differential correlation analysis involves a large number of pairwise tests relative to sample size, this lipid set-enrichment analysis is intended to identify exploratory network rewiring signatures rather than definitive causal interactions.

In addition to the exploratory network-based differential correlation approach, Graphical Lasso (GLasso) was also applied across lipidomic features. GLasso estimates a sparse precision matrix to ACS and CCS groups jointly and employs L1 (Lasso) regularization to find a parsimonious network where an edge between two lipids only survives if it cannot be explained by mutual correlations with other lipids [25]. This uniquely allows GLasso to estimate conditional independence, meaning that an edge in the GLasso network represents two lipids that are directly associated with one another after controlling for everything else in the network. While the differential correlation analysis performed does not guarantee conditional independence from the network, it is more robust for our dataset size constraints since GLasso requires more instances than features (n >> *p*). Therefore, GLasso is used only as a sensitivity analysis to corroborate the findings from differential correlation analysis after aggregation to the pathway level.

### 2.7. Machine Learning to Evaluate Predictive Capacity of Molecular Features

To evaluate the predictive value of molecular multi-omics over clinical traits alone, preliminary machine-learning models were trained and evaluated to predict ACS vs. CCS outcome. Eighteen lipid structural-class specific feature sets were compared against (1) clinical traits, including demographics, health history, and hematological features from a complete blood count (CBC) panel, and (2) the full set of lipidomic features. Cardiac troponin was not included as a predictor in machine-learning models because it forms part of the diagnostic criteria for ACS and therefore would introduce label leakage and circularity into the classification framework. The best performing lipid classes are discussed in the main manuscript; however, comprehensive model performance results can be found in the Supplementary Materials.

For each feature set, five model architectures were explored: logistic regression, decision tree, random forest classifier, adaptive boosted ensemble, and a support vector machine (SVM) with a linear kernel. Models were evaluated via 5-fold stratified cross validation to fairly represent model generalizability on this small patient cohort. Additionally, to avoid overfitting models, no hyperparameter tuning was conducted in this preliminary evaluation. The champion architecture is selected by the greatest mean area under the receiver operating characteristic (AUROC). Evaluation metrics include the out-of-fold AUROC and area under the precision-recall curve (AUPRC) and the sensitivity at fixed specificity thresholds of 90, 95, and 99%. The net reclassification index (NRI) is calculated for each feature set with respect to the clinical trait control feature set.

Model explainability is achieved by calculating permutation feature importance (PFI) scores as the change in mean AUROC from ablation of each feature in the feature set over the five validation set folds. The PFI scores for each feature are normalized to their feature set to account for biases in dimension size comparing different feature sets.

### 2.8. Statistical Environment and Significance Testing

Statistical analysis was performed using Python (version 3.10). Levene’s test revealed an abundance of molecules with non-homogenous variance between ACS and CCS patients, so Welch’s *t*-test was used over Student’s *t*-test to evaluate univariate associations. Univariate logistic regression models per lipid feature were initially adjusted with more anthropometric covariates; however, this resulted in overfit models due to a limited cohort size; therefore, age and sex were prioritized as covariates to adjust molecular effect size contributions to CAD outcome. All significance tests used a threshold of *p* < 0.05, with FDR adjustment for multiple hypothesis tests via Benjamini-Hochberg correction (*q* < 0.05). Any value less than this is regarded as statistically significant, and observations reliant on nominal associations are reported accordingly. Pipelines, figures, and anonymized and de-identified data are made available at https://github.com/vkanpa01/network-lipidomics-in-acute-coronary-syndrome.

## 3. Results

### 3.1. Blood Plasma Traits Demonstrate Significant Effect Size in ACS Patients Compared to CCS Patients

Univariate statistical tests on the CAD cohort reveal that ACS (*n* = 19) and CCS (*n* = 57) groups were well-matched on age (72.6 ± 12.1 vs. 69.6 ± 10.5 years; *p* = 0.34), sex (74% vs. 77% male; *p* = 0.76), BMI (27.5 ± 5.8 vs. 27.4 ± 3.7, *p* = 0.92), and prevalence of hypertension, diabetes, prior MI, and atrial fibrillation (all *p* > 0.2) (Table 1). Additionally, nominal differences were observed across red blood cell count (*p* = 0.002), hematocrit percentage (*p* = 0.0113), hemoglobin concentration (*p* = 0.0113), concentration of glutamate oxaloacetate transaminase (GOT)—more commonly known as aspartate aminotransferase (AST)—(*p* = 0.0212), obesity (*p* = 0.0336), and statin treatment (*p* = 0.015). However, after FDR correction, none of the complete blood count (CBC), anthropomorphic traits, or clinical comorbidities emerge as statistically significant (*q* > 0.05). Red blood cell count emerges as the closest to statistical significance, with *q* = 0.0635. This suggests that the two CAD subgroups are well-matched and lipidomic differences observed in subsequent analyses are not secondary to systematic clinical confounding.

### 3.2. Molecular Lipidomic Features Show Widespread Depletion in ACS Patients

**Figure 1:**
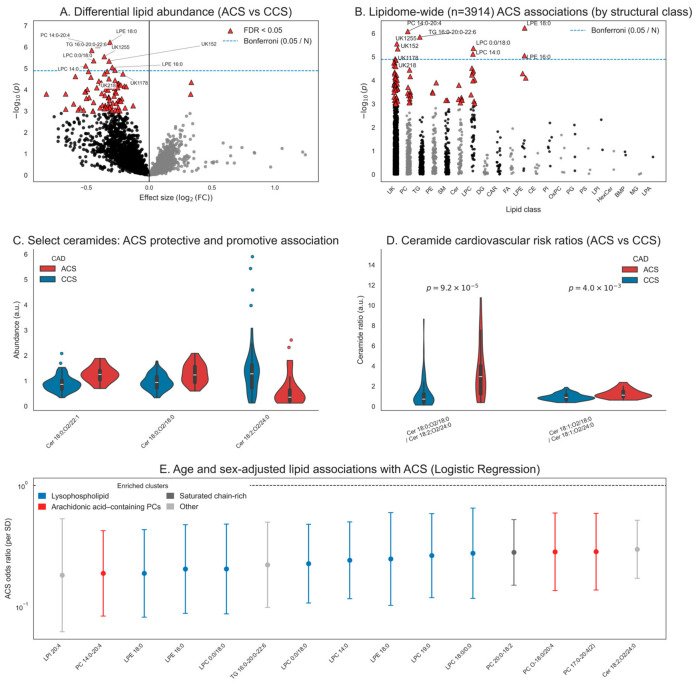
Differential lipid abundance between ACS and CCS. (**A**) Volcano plot of 3914 quantified platelet lipid features in ACS vs. CCS; significant lipids are shown as red triangles and a stringent Bonferroni threshold is shown in a blue line. (**B**) Manhattan plot of FDR-significant lipids by structural class. (**C**) Selected ceramide species with nominal positive associations with ACS, alongside Cer 18:2;O2/24:0 (strongest inverse association). (**D**) Selected ceramides expressed as a ratio to demonstrate non-linear ACS-specific relationships in ceramide abundance. (**E**) Age- and sex-adjusted logistic regression odds ratios for top FDR-significant lipids with OR < 1.0 for ACS. ORs shown with 95% CI. UK-prefixed identifiers denote unresolved structural annotations.

Out of 3914 molecular features from lipidomics data, 81 showed significant differences in mean abundance between ACS and CCS cohorts after Benjamini-Hochberg FDR correction (*q* < 0.05), and 9 remained significant even under a rigorous Bonferroni corrected threshold (*p* < 1.28 × 10^−5^) (Figure 1A). The structural classes with the greatest proportion of differentially expressed known lipids were phosphatidylcholine (*n* = 12; PC; 14:0-20:4, 14:0-20:5, 15:0-20:4, 17:0-18:1, 17:0-20:4, 20:0-18:2, 18:0-20:4, O-18:0/20:4, 18:2-20:4, 20:4-20:4, 20:0-20:4, 20:4-HDOHE), lysophosphatidylcholine (*n* = 9; LPC; 14:0, 0:0/16:0, 16:0/0:0, 17:0/0:0(1), 17:0/0:0(2), 18:0/0:0, 0:0/18:0, 19:0, 22:4), and ceramides (*n* = 6; Cer; 18:0;O2/22:1, 17:1;O2/22:0, 18:1;O2/23:0, 18:1;O2/22:0, 18:2;O2/24:0) (Figure 1B). The age- and sex-adjusted univariate logistic regression models fitted on each molecule estimated significant effect sizes for seven lipids (*q* < 0.05), of which five were structurally annotated molecules (Cer 18:2;O2/24:0, PC 14:0-20:4, PC 20:0-18:2, LPE 18:0, LPC 0:0/18:0) (Table 2). While no lipids had achieved elevated odds for ACS with statistical significance, increased Cer 18:0;O2/22:1 (*q* = 0.088, OR = 3.072 [1.607, 5.878]) and Cer 18:0;O2/18:0 (*q* = 0.088, OR = 2.791 [1.515, 5.145]) demonstrate nominally significant promotive effects on ACS (Figure 1C). Interestingly, Cer 18:2;O2/24:0 (*q* = 0.021, OR = 0.297 [0.171, 0.515]) demonstrates the most significant reduced likelihood of ACS out of all identifiable lipids.

To explore whether ceramide dysregulation in ACS could be captured as a composite ratio, we computed two ceramide ratios (Figure 1D). After examining univariate associations, a post-hoc ratio between Cer 18:0;O2/18:0 and Cer 18:2;O2/24:0 was 4.1-fold elevated in ACS compared to CCS (median 2.94 [IQR 1.25–4.01] vs. 0.72 [0.41–1.23]; Mann-Whitney *p* = 9.2 × 10^−5^). Interestingly, the numerator molecule (which is abundant in ACS) is a direct dihydroceramide precursor for the denominator molecule (which is depleted in ACS) (Table 2). A second ratio consistent with the published CERT2 ceramide cardiovascular risk score [26] (Cer 18:1;O2/18:0/Cer 18:1;O2/24:0) was also nominally significantly elevated in ACS (median 1.12 vs. 0.89; *p* = 0.004). All statistically significant molecules (*q* < 0.05) from univariate logistic regression had odds ratios for an ACS outcome of less than 1.0 (Figure 1E). None of the 63 targeted miRNAs exhibited significant differential expression between ACS and CCS (Supplementary Figure S1). A comprehensive table of *t*-statistics and logistic regression coefficients for lipidomic and miRNA analyses is available in the Supplementary Materials (Supplementary Table S1).

### 3.3. Differential Lipid Correlations Suggest ACS Rewiring Around Central Oxidized Lipids

**Figure 2:**
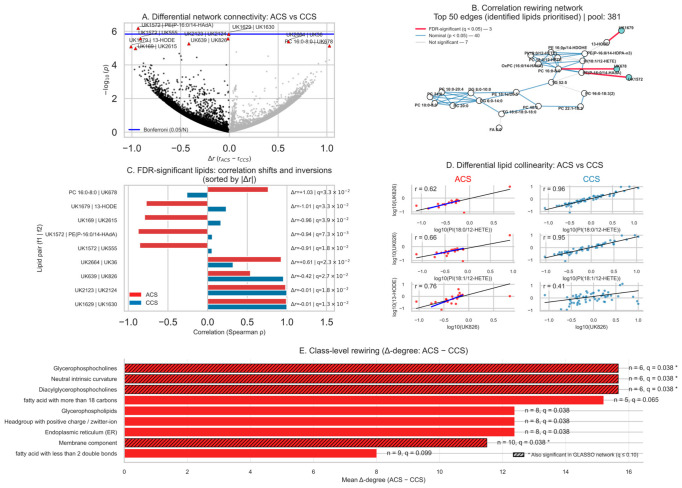
Differential lipid correlation analysis. (**A**) Distribution of change in Spearman correlation (Δr) between ACS and CCS across 34,191 lipid pairs. (**B**) Differential correlation network prioritizing the top 50 lipid pairs of identifiable molecules, by FDR-corrected q value. Three unidentifiable lipids are included because they achieve significant differential correlation with at least 1 identifiable lipid. (**C**) FDR-significant lipids demonstrating correlation shifts and inversions. (**D**) Differential correlation changes between ACS and CCS among oxidized lipids. Dark blue lines on the ACS panels represent the Theil-Sen best-fit line after excluding points beyond the 1.5 × IQR threshold on both axes, shown where ≥1 outlier was detected; the solid black line uses all points. (**E**) Enriched ontologies in ACS relative to CCS using identifiable lipids corresponding to nominally (*p* < 0.05) or FDR-significantly rewired lipid pairs. UK-prefixed identifiers denote unresolved structural annotations.

Among 34,191 lipidomics pairs of molecular features, 9 exhibited non-trivial significant differences in correlation strength between ACS and CCS (*q* < 0.05); an additional 3 pairs have perfect correlations in ACS (r_ACS_ = 1.0) and near-perfect correlations in CCS (r_CCS_ > 0.999), suggesting impractically significant differential correlations (Table 3). Additionally, 2104 pairs of lipid features achieve nominal significance (*p* < 0.05) (Figure 2A). A correlation rewiring network of statistically significant pairs (*q* < 0.05) reveals no central nodes corresponding to identifiable lipids. However, adding the top 50 nominally significant correlations between identifiable lipid species reveals a few central lipids, including PE(P-16:0/14-HDPA-n3), PC 20:0, PI(18:0/12-HETE), and PC 10:0-20:4 (Figure 2B), but adding the top 30 nominal relationships to this network reveals centrality of a few known species, including PE(P-16:0/14-HDPA-n3), Unknown(826), PI(18:0/12-HETE), and PC 10:0-20:4 (Supplementary Figure S3). Five pairs of molecules showed significant ACS correlation inversions: four pairs are positive in CCS and negative in ACS, and one pair is positive in ACS and negative in CCS (Figure 2C). Two of these pairs include known lipids 13-Hydroxyoctadecadienoic acid (13-HODE) (*q* = 0.032, Δr = −1.01) and PE (P-16:0/14-HDPA-n3) (*q* = 0.0073, Δr = −0.94). Most significant pairs involved unidentified lipids; however, in the second-largest subnetwork, Unknown (826) emerged as a hub connecting oxidized phosphatidylinositol (PI(18:0/12-HETE)) and oxidized phosphatidylethanolamine (PE(P-16:0/14-HDPA-n3)) (Figure 2D). While only a small subset of lipid pairs remained significant after FDR correction, the broader set of 2104 nominal hits was examined to generate hypotheses regarding class-level network reorganization. From the subset of identifiable lipids among these hits, significantly rewired ontology terms in ACS generally describe enrichment of phosphatidylcholine (PC)-like membrane lipids (Figure 2E). Enriched terms include glycerophosphocholine (*q* = 0.031), diacyl-PC (*q* = 0.031), neutral intrinsic curvature (*q* = 0.031), headgroup with positive charge (*q* = 0.081), and membrane component (*q* = 0.044) or ER (*q* = 0.081) localization. While these ontological terms are somewhat redundant, they suggest that the platelet membrane plays an important role in differentiating the severity of the disease. Moreover, enrichment of ER localization may be indicative of the role of the (dihydro)ceramides, which are de novo biosynthesized in ER. No features among the miRNA array achieved statistical significance (Supplementary Figure S2). Comprehensive statistics for differential correlation pairs among lipidomics and miRNA transcriptomic features are available in the Supplemental Materials (Supplementary Table S2).

### 3.4. Phosphatidylcholine (PC) and Ceramide Trained Models Outperform a Complete Blood Count-Derived Clinical Traits Benchmark in ACS Classification

**Figure 3:**
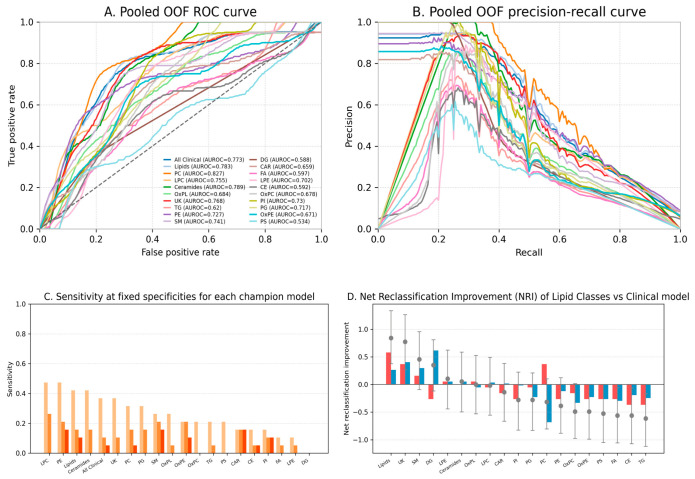
Cross-validated machine-learning performance. (**A**) Mean AUROC across five folds for lipid class–specific and control feature sets, rendered over 1000 bootstraps. (**B**) Mean AUPRC across feature sets. (**C**) Sensitivity at 90% and 95% specificity. (**D**) Net reclassification index (NRI) relative to clinical and hematological traits, showing mixed event and non-event reclassification.

In a machine-learning analysis of 18 lipid-class-specific feature sets, a lipidome wide feature set, and a feature set of routine clinical and hematological traits, logistic regression was the most commonly selected model over alternative non-linear architectures, regardless of dimension size. The benchmark clinical trait feature set achieved a pooled out-of-fold (OOF) AUROC and AUPRC of 0.7955 and 0.6528, respectively. The molecular features from lipidomics (positive control) achieved comparable performance, with an OOF AUROC of 0.7836 and OOF AUPRC of 0.6162. Among lipid structural class-trained models, phosphatidylcholines (PCs) resulted in the highest pooled AUROC of 0.8336 and an AUPRC of 0.6625, while ceramides also demonstrated comparable performance to the clinical trait control (Figure 3A,B). The ceramide-trained model also achieves stronger sensitivity at a fixed 90% specificity threshold (0.4211) than all feature sets except LPC (0.4737) (Figure 3C). Although oxidized phospholipids (OxPLs) emerge from prior network and differential expression analyses, they fit a modest model with no demonstration of significant classification improvement to the clinical trait model (Table 4).

The net reclassification index (NRI), relative to the clinical trait benchmark model, was calculated against notable lipid classes and the full lipidomic assay feature set (Figure 3D). On average, the lipidomic feature-trained model (NRI_total_ = 0.8421; 95% CI = [0.3731, 1.3335]) systematically moves both ACS predicted probabilities upward (NRI ACS event = 0.5789) and CCS predicted probabilities downward (NRI non-ACS event = 0.2632) compared to a clinical and hematological trait model. This indicates that a model trained on lipidomic features improves continuous risk scores determined from the clinical model. Ceramide, OxPL, and LPC-trained models show no compelling evidence of improved ranking of risk probabilities compared to clinical traits. However, the PC-trained model (NRI ACS event = 0.3684, NRI non-ACS event = −0.6842) has a total NRI of −0.3158 (95% CI = [−0.8044, 0.1011]), indicating that predicting ACS using PC pushes the clinical model’s risk assessment in the positive direction but also incorrectly raises CCS patients’ risk probabilities compared to the clinical model.

AUROC and AUPRC curves, sensitivity testing, NRI comparisons against a clinical feature set, and model calibration curves are shown across all lipid-specific and control feature sets in Supplementary Figure S4. Champion model performances on all lipid-specific and control feature sets are available in Supplementary Table S3.

**Figure 4:**
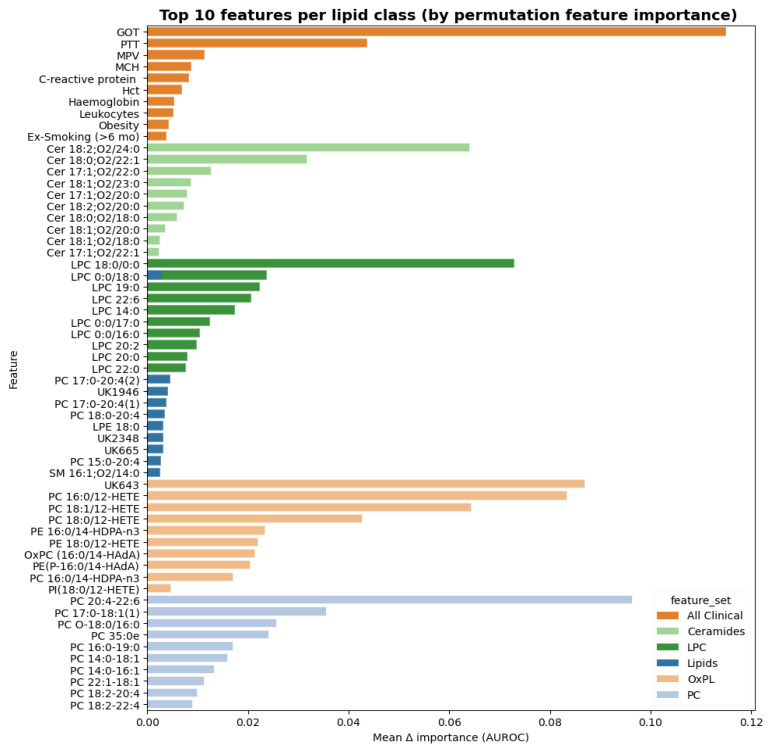
Normalized mean permutation feature importance (PFI), among features with a positive PFI, in champion models. Permutation feature importance is calculated as a change in mean AUROC from 5-fold cross validation following feature ablation. LPC 0:0/18:0 is selected by two models and is indicated accordingly with a stacked color encoding.

Feature ablation analysis reveals that in the clinical traits model, the liver enzyme GOT/AST contributed significantly more to prediction outcomes than all other complete blood count (CBC) traits (Figure 4). Five out of the 10 top-ranked molecules by normalized PFI in the lipidomics-trained champion model belonged to PC or LPC classes (LPC 0:0/18:0, PCs 15:0-20:4, 18:0-20:4, 17:0-20:4(1), and 17:0-20:4(2)). The most important ceramide for model predictions, Cer 18:2;O2/24:0, emerged previously with significant OR for CCS in the age- and sex-adjusted logistic regression analysis. Interestingly, it is one of the few ceramides whose depletion, not elevation, is significantly associated with ACS. Even among OxPLs, oxidized PCs demonstrate the strongest contribution to model predictions.

A comprehensive table of PFI scores across all models is available in the Supplementary Materials (Supplementary Table S4) and the top 10 features (if available) with positive mean PFI scores in each feature set are shown in Supplementary Figure S5.

## 4. Discussion

In this pilot study of patients with symptomatic coronary artery disease (CAD) undergoing coronary angiography, we integrated platelet lipidomic profiling with clinical variables to explore molecular differences between acute coronary syndromes (ACS) and chronic coronary syndromes (CCS). The central finding is that platelet lipidomics reveals biologically coherent and mechanistically interpretable lipid remodeling in ACS, with ceramide species emerging as the most specific and clinically translatable signal in this cohort. Several principal findings emerged. First, platelet lipidomics revealed widespread differences between ACS and CCS, with 81 lipid features significantly altered at FDR (*q* < 0.05), prominently involving phosphatidylcholines (PCs), lysophosphatidylcholines (LPCs), and ceramides. Second, differential correlation analysis suggested acute lipid network rewiring, with oxidized phospholipid species emerging as connectivity hubs. Third, in a cross-validated machine-learning benchmark for ACS classification, untargeted platelet lipidomics did not outperform a baseline model composed of routine clinical and hematological traits; however, models fitted on PCs demonstrated modest gains in AUROC, and the overall pattern of findings supports a targeted validation strategy centered on ceramide and phospholipid candidates. Together, these findings support a model in which acute platelet sphingolipid and phospholipid remodeling accompanies ACS, generating biologically plausible candidate biomarkers and mechanistic hypotheses while underscoring the need for targeted validation and external replication before clinical translation.

### 4.1. Platelet Membrane Phospholipid Remodeling in ACS

Our findings are consistent with growing evidence that the platelet lipidome is dynamically altered in symptomatic CAD and differs between ACS and CCS states. In a comprehensive platelet lipidomics study of symptomatic CAD patients, Harm and colleagues demonstrated substantial remodeling of glycerophospholipids in ACS compared with CCS, including shifts in specific PC species that were functionally linked to platelet activation and thrombotic potential [11]. Subsequent large-cohort work further supported the clinical relevance of platelet lipid signatures in symptomatic CAD, associating specific lipid patterns with ischemic outcomes [17]. Our observation that numerous PCs and LPCs were significantly reduced in ACS aligns directionally with this literature, suggesting that platelet membrane composition reflects activation state and thrombo-inflammatory burden.

During platelet activation, cytosolic phospholipase A2 (cPLA2)-mediated cleavage of membrane phospholipids generates lysophospholipids and releases polyunsaturated fatty acids (PUFAs) from PUFA-phospholipids (PUFA-PLs) that participate in eicosanoid synthesis and downstream signaling [27,28]. The observed decrease in PUFA-PCs should be interpreted as a reflection of cPLA2 activity, and the LPC depletion due to microvesicle formation and release through membrane shedding in response to apoptosis [29]. This consumption of membrane phospholipids is therefore consistent with a picture of heightened platelet activation in ACS. Additionally, the observed enrichment of PUFA-containing PC and LPC species among the most significantly depleted lipids supports a model in which arachidonic acid and other PUFA precursors are mobilized for eicosanoid and thromboxane synthesis during acute thromboinflammatory events. Evidence in cell and animal models and in human patients suggests polyunsaturated fatty acid (PUFA), which contains phospholipids (i.e various PE, PC, PI, and PS molecules) that are enriched in platelets during platelet production as a result of megakaryocyte differentiation [30].

### 4.2. Ceramide Remodeling and Platelet Sphingolipid Biology in ACS

A central and novel finding of this study is the specific pattern of ceramide remodeling observed in ACS platelets. Six ceramide species were significantly depleted in ACS after FDR correction, and Cer 18:2;O2/24:0 demonstrated the strongest inverse association with ACS of any identifiable lipid in the entire dataset (*q* = 0.021, OR = 0.297, 95% CI = [0.171, 0.515]). Notably, however, two ceramide species—Cer 18:0;O2/22:1 and Cer 18:0;O2/18:0—showed nominally significant positive associations with ACS (OR = 3.072 and 2.791, respectively), a divergence from the actual observed ceramide depletion that is unlikely to reflect analytical noise and warrants mechanistic interpretation.

Ceramides are bioactive sphingolipids generated primarily through two routes during platelet activation: de novo synthesis via ceramide synthases (CerS) in ER, and sphingomyelinase (SMase)-mediated hydrolysis of sphingomyelin at the platelet membrane [31]. Degranulation and membrane scrambling during platelet activation involve sphingomyelin phosphodiesterase-1 (SMPD1), which breaks down sphingomyelin to ceramide [31]; however, the depletion of most ceramide species observed here in ACS cohort platelets may reflect rapid downstream catabolism rather than reduced production. Specifically, the ceramide to sphingosine to sphingosine-1-phosphate (S1P) pathway is well-characterized during platelet activation. Platelet activation causes ceramide breakdown with concurrent increases in sphingosine and S1P release [29], and S1P is stored abundantly in platelets and released upon degranulation as a potent platelet activator and vasoactive mediator [17,32]. Therefore, ceramide consumption through this catabolic S1P route is consistent with the widespread depletion pattern observed in this ACS cohort.

The two ceramide species with nominally elevated ACS signal—Cer 18:0;O2/22:1 and Cer 18:0;O2/18:0—differ from the depleted species in fatty acid chain length and degree of unsaturation. This divergence may reflect on one hand increased biosynthesis of dihydroceramides in the ER (e.g., Cer 18:0;O2/18:0), and, on the other hand, differential CerS isoform activity during activation and/or apoptosis, as CerS isoforms (CerS1–CerS6), exhibits distinct acyl chain-length specificities and is differentially expressed and regulated across activation states [33]. Ceramide chain length-specific functions and associations with cardiovascular disease have increasingly been recognized in numerous clinical contexts [33,34]. The bidirectional ceramide signal observed here (coupling a simultaneous depletion of certain ceramide pools and an apparent accumulation via distinct synthetic routes) is a hypothesis that could be addressed in future mechanistic platelet activation experiments with isoform-specific SMase inhibitors and CerS substrates.

Critically, this ceramide signal is platelet-specific and therefore is likely mechanistically distinct from what would be captured by plasma lipidomics. Plasma ceramide elevations in cardiovascular disease reflect systemic lipid metabolism and are associated with plaque vulnerability, inflammation, and atherothrombotic processes [34,35,36,37]. Specifically, in ACS, platelet ceramide dynamics reflect the acute thromboinflammatory activation state of the platelet itself. This distinction crucially matters for both mechanistic interpretation and translational strategy, since the development of ceramide-based cardiovascular risk scores (beginning from discovery lipidomics through the CERT and CERT2 validated panels [26,34,37,38,39]) demonstrates a pathway for how ceramide candidates identified in untargeted studies can be refined into analytically robust, clinically deployable tools. The CERT/CERT2 panels were derived from plasma, not platelets, and validated for chronic cardiovascular risk prediction [26,37]. By contrast, a platelet-specific ceramide panel would target the acute thromboinflammatory biology of ACS and could offer mechanistic information complementary to, rather than redundant with, existing plasma-based risk scores. These results suggest that a platelet lipid panel targeting select ceramide species, alongside candidate PC, LPC, and OxPL molecules identified here, represents the most realistic and scientifically grounded translational step from this cohort, and that such a panel should be ceramide-enriched based on the specificity and effect size of the ceramide signal observed. Importantly, the platelet lipid signatures identified in this study are not intended as alternative diagnostic biomarkers but rather as candidate markers that may complement existing clinical tools. Indeed, plasma lipidomic signatures have previously been used in the risk stratification of patients with cardiovascular disease, diabetes, and sepsis [40,41,42]. Therefore, the greatest potential of platelet lipidomic signatures may lie in improving cardiovascular risk stratification in a targeted manner, identifying biologically distinct patient subgroups while also providing mechanistic insight into disease processes.

### 4.3. Oxidized Phospholipid Network Rewiring in ACS

Differential correlation analysis identified a pattern of lipid network rewiring between ACS and CCS cohorts. Among 34,191 lipidomic feature pairs, 9 exhibited significant differences in correlation strength after FDR correction, with an additional 2104 pairs achieving nominal significance. A rewiring network incorporating the top nominal relationships between identifiable lipid species revealed centrality of oxidized lipid species, including PE(P-16:0/14-HDPA-n3) and PI(18:0/12-HETE), alongside 13-HODE, a hydroxy-fatty acid resulting from linoleic acid oxidation. In the context of ACS-specific biology, these species are not spurious hits. Oxidized phospholipids (OxPLs) are bioactive mediators that support calcium-dependent coagulation factor binding, modulate platelet–endothelial interactions, and contribute to thrombo-inflammatory signaling [43]. Their emergence as platelet lipidome connectivity hubs in ACS, rather than simply being differentially abundant in ACS, suggests that the lipid network undergoes qualitative reorganization in the acute setting, with OxPL species gaining relational centrality that is not apparent from abundance data alone. This unique finding cannot be captured by standard differential abundance approaches and represents the methodological contribution of differential correlation analysis to this cohort’s dataset. The biological relevance of OxPLs in ACS is further supported by emerging evidence linking lipoprotein(a)-associated OxPLs to acute cardiovascular events and inflammatory signaling [44]. We acknowledge that the majority of significant differential correlation pairs involve unidentified features, and that the hub species identified here are hypothesis-generating rather than confirmed network nodes; however, the enrichment of identifiable OxPL species among these hubs is consistent with a biologically coherent interpretation. Moreover, the decrease of PUFA-PLs in ACS aligns with observed detection of various oxidized PUFA-PLs.

### 4.4. Machine-Learning Benchmarking: Scope, Comparator, and Translational Implications

In a machine-learning analysis of lipid class-specific feature sets and control groups, logistic regression was the most commonly selected model architecture regardless of feature set dimension. AdaBoost fitted with phosphatidylcholines (PCs) achieved the highest pooled AUROC (0.8273) and AUPRC (0.5683), modestly exceeding the clinical and hematological trait benchmark model (AUROC = 0.7733, AUPRC = 0.5291). The ceramide-trained RandomForest model also achieves comparable performance to the benchmark (AUROC = 0.7895, AUPRC = 0.5109).

High-sensitivity cardiac troponin, the canonical ACS biomarker and cornerstone of contemporary chest pain evaluation pathways [45,46] were deliberately excluded from all models because it contributes directly to the outcome definition, and its inclusion would introduce label leakage and circular classification. Future benchmarking studies should employ troponin-excluded clinical risk scores (such as HEART or GRACE) as the reference comparator, which would provide a more clinically meaningful and translationally rigorous baseline.

These findings do not diminish the biological relevance of platelet lipid remodeling; rather, they clarify the translational landscape. Untargeted multi-omics discovery platforms are optimized for mechanistic insight and candidate identification, whereas clinical deployment typically requires low-cost, analytically robust targeted panels. To this extent, the net reclassification analysis is instructive: for instance, the PC-trained model dramatically improves ACS event risk ranking, but worsens non-event ranking, yielding a negative total NRI. Given the class imbalance between ACS and CCS, AUROC values can remain stable despite misclassification of several CCS patients; however, NRI is very sensitive to these classification shifts. This indicates that ACS risk scoring is sensitive to changes in abundance of untargeted PC features, which yields higher risk scores for CCS events, as well when compared against a clinical trait benchmark model. This pattern is characteristic of high-dimensional discovery data applied to small-sample classification tasks (*p* >> n datasets) but may also reflect the genuine biological overlap between ACS and CCS, suggesting that some lipidomic features capture underlying coronary artery disease burden rather than acute plaque destabilization. The signal is real but insufficiently refined and validated for reliable clinical deployment. These discovery findings, particularly the ceramide and PC candidates identified here, should be used to design a targeted, hypothesis-driven panel for prospective validation in an adequately powered cohort. As aforementioned, the trajectory of ceramide-based risk scores (i.e CERT/CERT2) illustrates this pathway from discovery to clinically validated small panels [36,37,38].

### 4.5. Limitations and Future Directions

This study possesses several limitations that warrant consideration. First, this was a pilot study with 19 ACS events, limiting statistical power and increasing the risk of overfitting in high-dimensional modeling [47]. The machine-learning analyses should be interpreted in light of the modest sample size relative to the dimensionality of the dataset (>100 features in some lipid class models). Although cross-validation and within-fold preprocessing were implemented to mitigate bias, performance estimates and permutation feature importance rankings remain susceptible to instability unstable in small samples, particularly with correlated predictors. Notably, however, most cardiovascular lipidomic studies rely on targeted panels comprising a limited number of predefined lipids, which reduces feature dimensionality but constrains discovery. By contrast, our untargeted mass spectrometry-based approach enabled the identification of novel lipid candidates that would not be captured by targeted assays. Accordingly, the identified lipid signatures should be considered hypothesis-generating candidates that require validation in larger independent cohorts as highlighted by reporting and bias assessment frameworks such as TRIPOD+AI and PROBAST+ [48,49]. Second, sampling occurred at the time of angiography, limiting generalizability to early emergency department triage contexts. Third, cross-sectional design precludes causal inference or temporal dynamics, a point especially noteworthy for understanding the differential correlation analysis. Fourth, the control group comparator (patient clinical and hematological traits) does not represent conventional ACS diagnostic traits, which often include an ECG readout or troponin measurement; the machine-learning benchmark should be interpreted accordingly, as discussed above.

Platelet miRNA transcriptomics, collected and assessed as an exploratory arm of this study, yielded no significant discriminatory signal between ACS and CCS cohorts, neither in differential expression nor in differential correlation analyses. The cardiovascular-focused miRNA array used in this study quantified 63 pre-selected miRNAs with established or proposed relevance to cardiovascular biology. While this targeted approach enabled focused interrogation of biologically relevant candidates, the limited number of assayed miRNAs necessarily restricted transcriptomic coverage compared with unbiased small RNA sequencing approaches. Consequently, the absence of significant findings may reflect a combination of limited statistical power, the restricted breadth of the platform, the possibility that platelet miRNA differences between ACS and CCS are modest relative to inter-individual variability, or genuine biological absence of discriminatory miRNA signal in the platelet compartment under these sampling conditions. Platelet miRNA dynamics in ACS remains an area of active investigation [50,51,52,53,54], and the question merits revisitation in a prospectively powered study with more comprehensive transcriptomic coverage.

In the lipidomics analyses, we included data corresponding to unknown features, for which no structure could be assigned in the automated MS-DIAL peak picking, alignment, deconvolution, and structural annotation workflow. The motivation for this workflow was to annotate de novo unknown features that emerge from machine-learning data processing as significant or of interest. Retrospectively, it has to be admitted that this strategy did not yield great success, as only a handful of the unknown features could be finally fully annotated (e.g., oxidized phospholipids). For the majority of unknown features, the de novo structure elucidation procedure was ultimately unsuccessful, as many of them were close to the detection limit, lacking MS2 data, or were of other poor quality. Hence, the usefulness of inclusion of unknowns for data processing has to be critically assessed in future clinical campaigns.

Future studies should prioritize: (i) prospective validation of a targeted platelet ceramide and phospholipid panel in larger, independent cohorts; (ii) paired plasma and platelet lipid profiling to clarify compartment-specific dynamics and determine whether platelet ceramide signals offer information incremental to established plasma ceramide risk scores; (iii) mechanistic platelet activation experiments to define the ceramide remodeling-associated metabolic trajectories, including SMase activity, CerS isoform expression, and S1P generation; and (iv) longitudinal sampling in stable CAD patients to assess whether platelet lipid profiles predict incident ACS, distinguishing diagnostic from prognostic utility.

## 5. Conclusions

In summary, this pilot study leverages untargeted platelet lipidomics to reveal biologically coherent and mechanistically interpretable lipid remodeling in ACS compared with CCS. We observe widespread phosphatidylcholine and lysophosphatidylcholine depletion, which is consistent with heightened cPLA_2_ activity and membrane remodeling. Additionally, a bidirectional ceramide shift implicates differential sphingomyelinase and ceramide synthase activity. Furthermore, oxidized phospholipid species emerge as connectivity hubs in differential correlation analysis, a pattern not detectable by standard differential abundance approaches alone. The ceramide signal was notable for its specificity and effect size, with Cer 18:2;O2/24:0 demonstrating the strongest inverse association with ACS of any identifiable lipid, and two dihydroceramide species showing nominally elevated ACS associations. Together, this suggests acute thromboinflammatory ceramide remodeling, distinct from the systemic ceramide dynamics captured by previous plasma-based risk scores. Although untargeted platelet lipidomics did not meaningfully outperform a routine clinical and hematological trait machine-learning benchmark in cross-validated ACS classification, this finding clarifies the translational landscape and reinforces the utility of high-dimensional discovery data in small-sample settings for candidate identification. The trajectory of plasma ceramide risk scores from untargeted discovery to clinically robust targeted panels, exemplified by the CERT/CERT2 pathway, illustrates the appropriate next step for this cohort’s findings. Future work should prioritize prospective validation of a targeted platelet ceramide and phospholipid panel in independent cohorts, paired plasma-platelet profiling to establish compartment-specific predictive value, and mechanistic platelet activation experiments to elucidate the ceramide remodeling trajectories identified here.

## Figures and Tables

**Table 1:** Baseline clinical and demographic characteristics of the ACS and CCS cohorts. Characteristics are shown for patients with acute coronary syndrome (ACS; *n* = 19) and chronic coronary syndrome (CCS; *n* = 57). Continuous variables are compared using Welch’s two-sample *t*-test, while binary variables are compared using Fisher’s exact test.

Variable	Type	ACS (*n* = 19)	CCS (*n* = 57)	*p*-Value	FDR *q*-Value	Test
Age (years)	Continuous	72.63 ± 12.10	69.61 ± 10.49	0.3394	0.7291	Welch *t*-test
BMI (kg/m^2^)	Continuous	27.54 ± 5.76	27.37 ± 3.72	0.9161	1	Welch *t*-test
Red blood cell count (×10^6^/uL)	Continuous	3.63 ± 0.94	4.43 ± 0.51	0.002	0.0635	Welch *t*-test
Haematocrit (%)	Continuous	31.84 ± 8.22	37.47 ± 6.12	0.0113	0.1211	Welch *t*-test
Haemoglobin (g/dL)	Continuous	11.58 ± 2.33	13.66 ± 4.41	0.0113	0.1211	Welch *t*-test
MCH (pg)	Continuous	30.25 ± 2.13	29.26 ± 4.05	0.1847	0.5389	Welch *t*-test
MCHC (g/dL)	Continuous	34.49 ± 1.22	34.69 ± 1.23	0.538	0.7485	Welch *t*-test
MCV (fL)	Continuous	87.65 ± 4.96	85.90 ± 4.55	0.1853	0.5389	Welch *t*-test
RDW (%)	Continuous	14.01 ± 1.71	13.34 ± 0.95	0.1154	0.4688	Welch *t*-test
MPV (fL)	Continuous	10.19 ± 0.67	10.47 ± 0.83	0.1603	0.5389	Welch *t*-test
Platelets (×10^3^/uL)	Continuous	227.32 ± 81.10	223.73 ± 59.97	0.861	1	Welch *t*-test
Platelet count (×10^6^)	Continuous	243.16 ± 119.27	223.93 ± 95.48	0.5291	0.7485	Welch *t*-test
Leukocytes (×10^3^/uL)	Continuous	8534.74 ± 3926.93	7556.25 ± 2114.30	0.3115	0.7291	Welch *t*-test
CRP (mg/L)	Continuous	4.49 ± 6.83	1.30 ± 3.83	0.0658	0.3507	Welch *t*-test
Creatinine (mg/dL)	Continuous	1.02 ± 0.34	1.17 ± 1.02	0.3418	0.7291	Welch *t*-test
eGFR/MDRD (mL/min/1.73 m^2^)	Continuous	78.12 ± 43.55	71.28 ± 22.72	0.519	0.7485	Welch *t*-test
INR	Continuous	1.13 ± 0.25	1.08 ± 0.23	0.4394	0.7485	Welch *t*-test
PTT (s)	Continuous	38.05 ± 34.09	25.14 ± 5.25	0.1172	0.4688	Welch *t*-test
AST/GOT (U/L)	Continuous	44.06 ± 37.84	21.26 ± 9.32	0.0212	0.1692	Welch *t*-test
ALT/GPT (U/L)	Continuous	35.21 ± 47.51	27.80 ± 28.41	0.5275	0.7485	Welch *t*-test
LDH (U/L)	Continuous	196.26 ± 63.80	191.77 ± 40.28	0.7757	0.9547	Welch *t*-test
Male sex, n (%)	Binary	14 (74%)	44 (77%)	0.762	0.9547	Fisher exact
Arterial hypertension, n (%)	Binary	15 (79%)	45 (79%)	1	1	Fisher exact
Hyperlipidaemia, n (%)	Binary	7 (37%)	29 (51%)	0.4267	0.7485	Fisher exact
Diabetes mellitus, n (%)	Binary	6 (32%)	17 (30%)	1	1	Fisher exact
Current smoker, n (%)	Binary	3 (16%)	13 (23%)	0.7469	0.9547	Fisher exact
Ex-smoker (>6 months), n (%)	Binary	2 (11%)	8 (14%)	1	1	Fisher exact
Obesity, n (%)	Binary	6 (32%)	35 (61%)	0.0336	0.215	Fisher exact
Atrial fibrillation, n (%)	Binary	5 (26%)	11 (19%)	0.5278	0.7485	Fisher exact
Previous CABG, n (%)	Binary	2 (11%)	8 (14%)	1	1	Fisher exact
Previous MI, n (%)	Binary	2 (11%)	15 (26%)	0.2105	0.5614	Fisher exact
Chronic kidney disease, n (%)	Binary	4 (21%)	7 (12%)	0.4518	0.7485	Fisher exact
Aspirin, n (%)	Binary	9 (47%)	33 (58%)	0.439	0.753	Fisher exact
Clopidogrel, n (%)	Binary	0 (%)	8 (14%)	0.189	0.753	Fisher exact
Prasugrel, n (%)	Binary	0 (%)	4 (7%)	0.567	0.798	Fisher exact
Ticagrelor, n (%)	Binary	1 (5%)	9 (16%)	0.436	0.753	Fisher exact
Oral Anticoagulant, n (%)	Binary			0.063	0.375	Fisher exact
Warfarin		3 (16%)	2 (4%)			
DOAC		1 (5%)	12 (21%)			
ACE Inhibitor, n (%)	Binary	9 (47%)	27 (47%)	1	1	Fisher exact
ARB, n (%)	Binary	5 (26%)	18 (32%)	0.778	0.850	Fisher exact
Aldosterone Agonist, n (%)	Binary	2 (10%)	14 (25%)	0.330	0.753	Fisher exact
Diuretic, n (%)	Binary	10 (53%)	25 (44%)	0.599	0.798	Fisher exact
Ca-channel blocker, n (%)	Binary	3 (16%)	16 (28%)	0.369	0.753	Fisher exact
β-blocker, n (%)	Binary	12 (63%)	39 (68%)	0.779	0.850	Fisher exact
Statin, n (%)	Binary	10 (53%)	47 (82%)	0.015	0.176	Fisher exact

**Table 2:** Top differentially abundant lipid species in ACS versus CCS. Among the 25 most significantly differentially abundant lipid species, this table presents all 10 identifiable molecules, ranked by Benjamini–Hochberg FDR-corrected *q*-value from a two-sample *t*-test (*n* = 19 ACS, *n* = 57 CCS). Significant FDR-adjusted *q*-values are annotated with an asterisk. This list is supplemented by all statistically significant ceramide species not already represented in the top 25. Log fold-change is expressed as ACS/CCS. Age- and sex-adjusted odds ratios (OR) with 95% confidence intervals were estimated by logistic regression; lipids with significant ORs after FDR correction are noted with an asterisk. Mean values refer to normalized data obtained by a combination of SERRF and RUV random normalization methods.

Lipid Species	ACS Mean ± SD	CCS Mean ± SD	log2(FC)	*t*-Statistic	FDR *q*-Value	Adjusted OR [95% CI]	Adjusted FDR *q*
LPE 18:0	0.589 ± 0.082	0.731 ± 0.117	−0.312	−5.826	0.0016	0.19 [0.08, 0.43]	0.0460 *
PC 14:0-20:4	0.544 ± 0.105	0.721 ± 0.147	−0.408	−5.749	0.0016	0.19 [0.08, 0.42]	0.0460 *
TG 16:0-20:0-22:6	0.555 ± 0.115	0.762 ± 0.211	−0.456	−5.381	0.0018	0.22 [0.10, 0.50]	0.0654
LPC 0:0/18:0	0.547 ± 0.131	0.742 ± 0.145	−0.441	−5.469	0.0029	0.23 [0.11, 0.48]	0.0474 *
LPC 14:0	0.528 ± 0.149	0.749 ± 0.199	−0.505	−5.12	0.0042	0.24 [0.12, 0.50]	0.0577
LPE 16:0	0.599 ± 0.086	0.734 ± 0.146	−0.294	−4.912	0.0044	0.20 [0.09, 0.47]	0.0654
LPC 18:0/0:0	0.582 ± 0.109	0.727 ± 0.128	−0.32	−4.777	0.0074	0.34 [0.18, 0.63]	0.0804
PC 17:0-20:4(2)	0.595 ± 0.093	0.719 ± 0.117	−0.272	−4.671	0.0083	0.28 [0.14, 0.59]	0.0804
LPC 19:0	0.573 ± 0.140	0.756 ± 0.173	−0.4	−4.651	0.0086	0.26 [0.12, 0.59]	0.0903
PC O-18:0/20:4	0.616 ± 0.070	0.707 ± 0.098	−0.198	−4.404	0.0106	0.28 [0.14, 0.59]	0.0804
Cer 18:0;O2/22:1	0.806 ± 0.143	0.641 ± 0.157	0.33	4.25	0.0152	3.07 [1.61, 5.88]	0.0804
Cer 17:1;O2/22:0	0.554 ± 0.146	0.706 ± 0.164	−0.35	−3.816	0.0345	0.35 [0.17, 0.68]	0.1134
Cer 18:1;O2/23:0	0.570 ± 0.132	0.711 ± 0.173	−0.318	−3.702	0.0387	0.40 [0.22, 0.76]	0.1365
Cer 18:0;O2/18:0	0.811 ± 0.168	0.660 ± 0.156	0.297	3.451	0.0664	2.79 [1.52, 5.14]	0.1322
Cer 18:2;O2/24:0	0.599 ± 0.189	0.786 ± 0.175	−0.391	−3.794	0.0394	0.30 [0.17, 0.52]	0.0208 *

**Table 3:** Differentially co-expressed lipid pairs between ACS and CCS. Pairwise Spearman correlations were computed separately within the ACS and CCS groups for all lipidomic features. Differential co-expression was quantified using Fisher’s Z-transformation of the correlation difference (Δz = z_ACS_ − z_CCS_), with FDR correction by Benjamini–Hochberg across all 34,191 tested pairs. The table presents all 12 FDR-significant pairs (*q* < 0.05) and an additional 5 pairs that pass a looser significance threshold (*q* < 0.10). Δr = r_ACS_ − r_CCS_. The direction of each rewiring event is classified as: inverted (sign change between groups), strengthened (same sign, |r_ACS_| > |r_CCS_|), or weakened (same sign, |r_ACS_| < |r_CCS_|). UK-prefixed identifiers denote unresolved structural annotations.

Partner 1	Partner 2	r (ACS)	r (CCS)	Δr (ACS–CCS)	Δz Statistic	*p*-Value	FDR *q*-Value	Direction
UK1059	UK1058	1	0.9998	0.0002	9.2579	0.00	0.0000	strengthened
UK1649	UK1648	1	0.9994	0.0006	11.3732	0.00	0.0000	strengthened
UK672	UK671	1	0.9984	0.0016	12.9836	0.00	0.0000	strengthened
UK1572	PE(P-16:0/14-HAdA)	−0.8754	0.0602	−0.9357	−4.9754	6.51 × 10^−7^	0.0073	inverted
UK1629	UK1630	0.993	0.9995	−0.0066	−4.8166	1.46 × 10^−6^	0.0131	weakened
UK1572	UK555	−0.8561	0.0567	−0.9129	−4.6919	2.71 × 10^−6^	0.0175	inverted
UK2123	UK2124	0.9842	0.9989	−0.0147	−4.6896	2.74 × 10^−6^	0.0175	weakened
UK2664	UK36	0.9281	0.3216	0.6064	4.6053	4.12 × 10^−6^	0.0231	strengthened
UK639	UK826	0.5404	0.9561	−0.4158	−4.546	5.47 × 10^−6^	0.0273	weakened
PC 16:0-8:0	UK678	0.7649	−0.2619	1.0268	4.4831	7.36 × 10^−6^	0.0330	inverted
UK1679	13-HODE	−0.7737	0.2359	−1.0096	−4.4612	8.15 × 10^−6^	0.0332	inverted
UK169	UK2615	−0.7947	0.1683	−0.9631	−4.406	1.05 × 10^−5^	0.0394	inverted
UK1159	UK1415	0.993	0.9219	0.0711	4.2966	1.73 × 10^−5^	0.0598	strengthened
UK2802	UK826	0.414	0.929	−0.5149	−4.252	2.12 × 10^−5^	0.0679	weakened
PE(P-16:0/14-HDPA-n3)	UK2802	0.4316	0.9288	−0.4973	−4.1736	3.00 × 10^−5^	0.0848	weakened
PC 10:0-8:0	PC 10:0-20:4	0.9474	0.5498	0.3976	4.1716	3.02 × 10^−5^	0.0848	strengthened
PI(18:0/12-HETE)	UK826	0.6211	0.9565	−0.3355	−4.1331	3.58 × 10^−5^	0.0944	weakened

**Table 4:** Machine learning model performance across lipidomic feature sets. Performance metrics are reported for each feature set’s champion model, which is defined as the model architecture achieving the highest mean AUROC. Performance metrics reflect the aggregate out-of-fold (OOF) AUROC and AUPRC from 5-fold cross-validation. Sensitivity at fixed specificity thresholds (90% and 95%) was computed from pooled OOF predictions using the operating point on the ROC curve that maximizes sensitivity while maintaining the target specificity. The Net Reclassification Improvement (NRI) is the continuous NRI computed against the clinical and hematological (“All Clinical”) reference model; 95% confidence intervals were derived from 1000 bootstrap resamples of the pooled out-of-fold predictions.

Feature Set	Champion Model	OOF AUROC	OOF AUPRC	Sensitivity @ 90% Specificity	Sensitivity @ 95% Specificity	NRI vs. All Clinical [95% CI]
All Clinical	RandomForest	0.7733	0.5291	0.3684	0.1053	*Reference*
Lipids	LogisticRegression	0.783	0.5685	0.4211	0.1579	0.8421 [0.3731, 1.3335]
PC	AdaBoost	0.8273	0.5683	0.3158	0.1579	−0.3158 [−0.8044, 0.1011]
LPC	RandomForest	0.7548	0.4992	0.4737	0.2632	−0.0175 [−0.5556, 0.4899]
Ceramides	RandomForest	0.7895	0.5109	0.4211	0.1579	0.0526 [−0.4958, 0.5863]
OxPL	LogisticRegression	0.6842	0.403	0.2632	0.0526	0 [−0.5326, 0.5264]

## Data Availability

Pipelines, figures, and anonymized and de-identified data are made available at https://github.com/vkanpa01/network-lipidomics-in-acute-coronary-syndrome. The data presented in this study are available on request from the corresponding authors.

## Supplementary Materials

The following supporting information can be downloaded at: https://www.mdpi.com/article/10.3390/cells15131190/s1, Figure S1: Differential miRNA abundance in ACS vs. CCS subtypes; Figure S2: Differential miRNA correlation analysis: ACS vs. CCS; Figure S3: Expanded lipid correlation rewiring network; Figure S4: ML modeling performance across all lipid classes, with calibration curves for champion models; Figure S5: Top predictive features by model (permutation importance) Table S1: Lipidomic and transcriptomic univariate significance tests and logistic regression modeling results; Table S2: Differential correlation pairs among lipidomics and miRNA transcriptomic features; Table S3: Champion model performances on all lipid-specific and control feature sets; Table S4: Permutation feature importance scores across all lipid-class machine learning models.
