# Supplementary figures and images for: Identifying Platelet Lipidomic Networks and Evaluating Machine-Learning Models to Identify Distinctive Features Between Chronic and Acute Coronary Syndrome
Source: Cells. 2026 Jun 30;15(13):1190. doi: 10.3390/cells15131190 (PMC13359697; doi:10.3390/cells15131190)

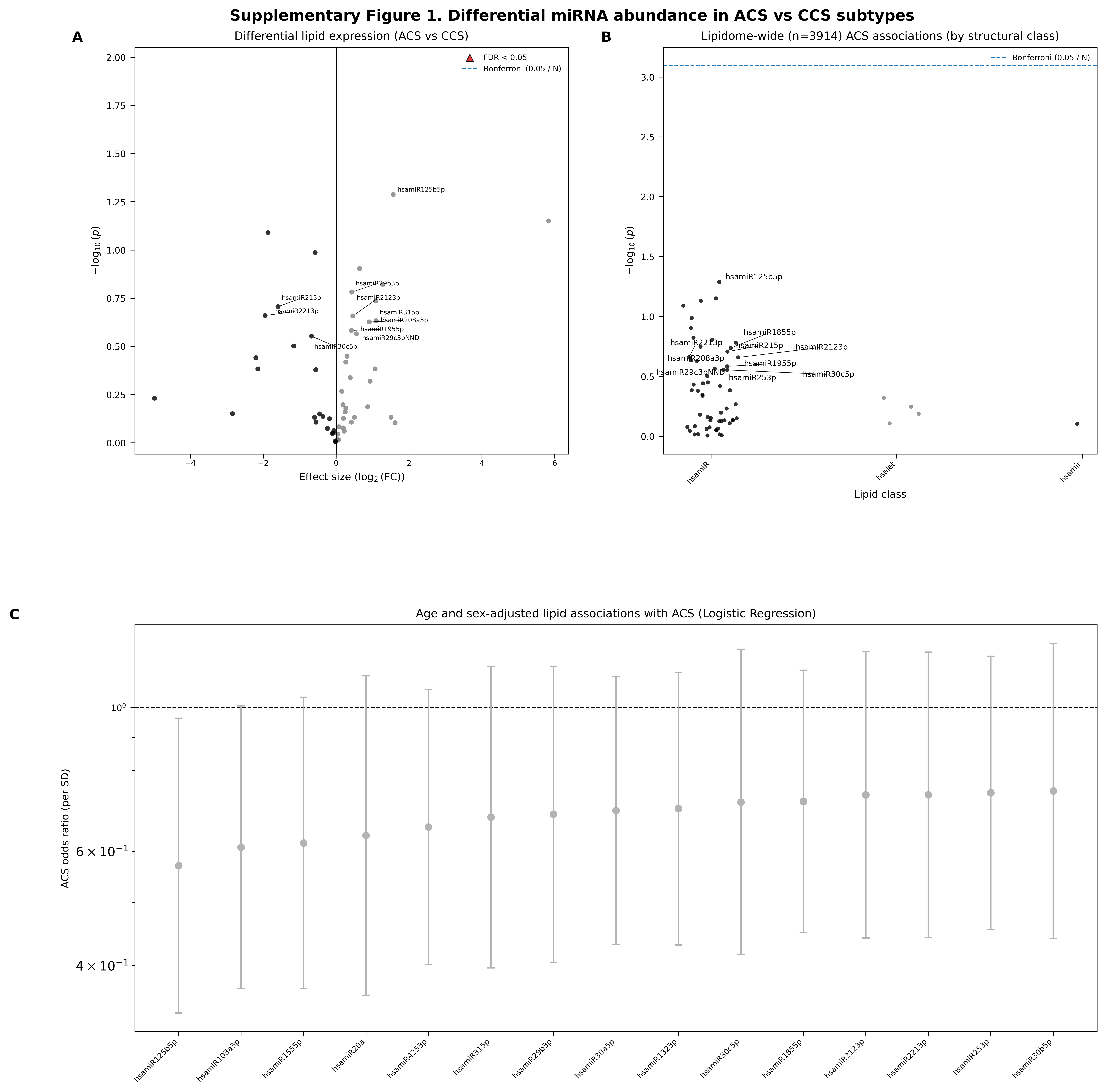

Supplement: Supplementary file 1 [file cells-15-01190-s001.zip › Kanpa_2026_Supplemental_Figure S1.png]

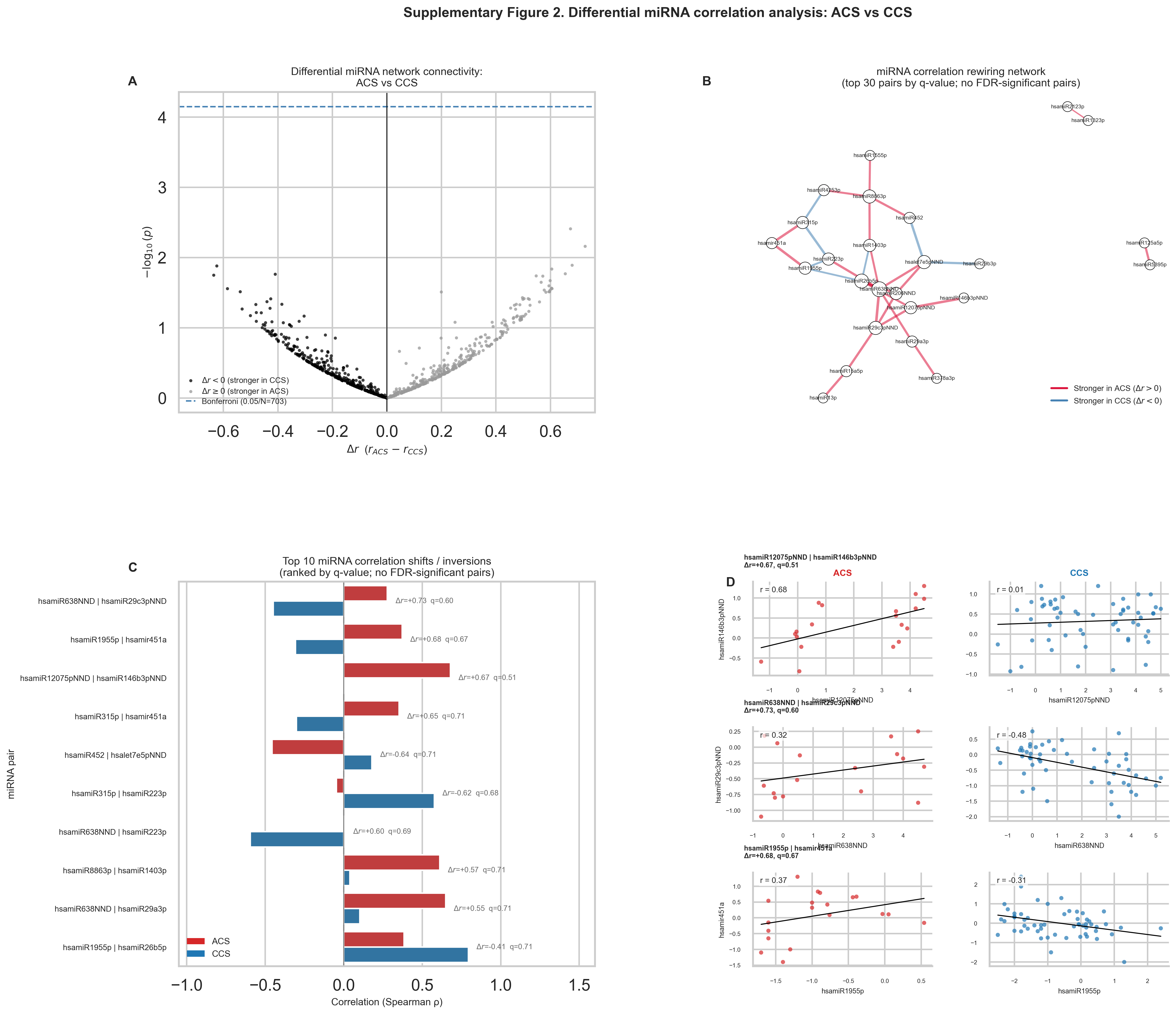

Supplement: Supplementary file 1 [file cells-15-01190-s001.zip › Kanpa_2026_Supplemental_Figure S2.png]

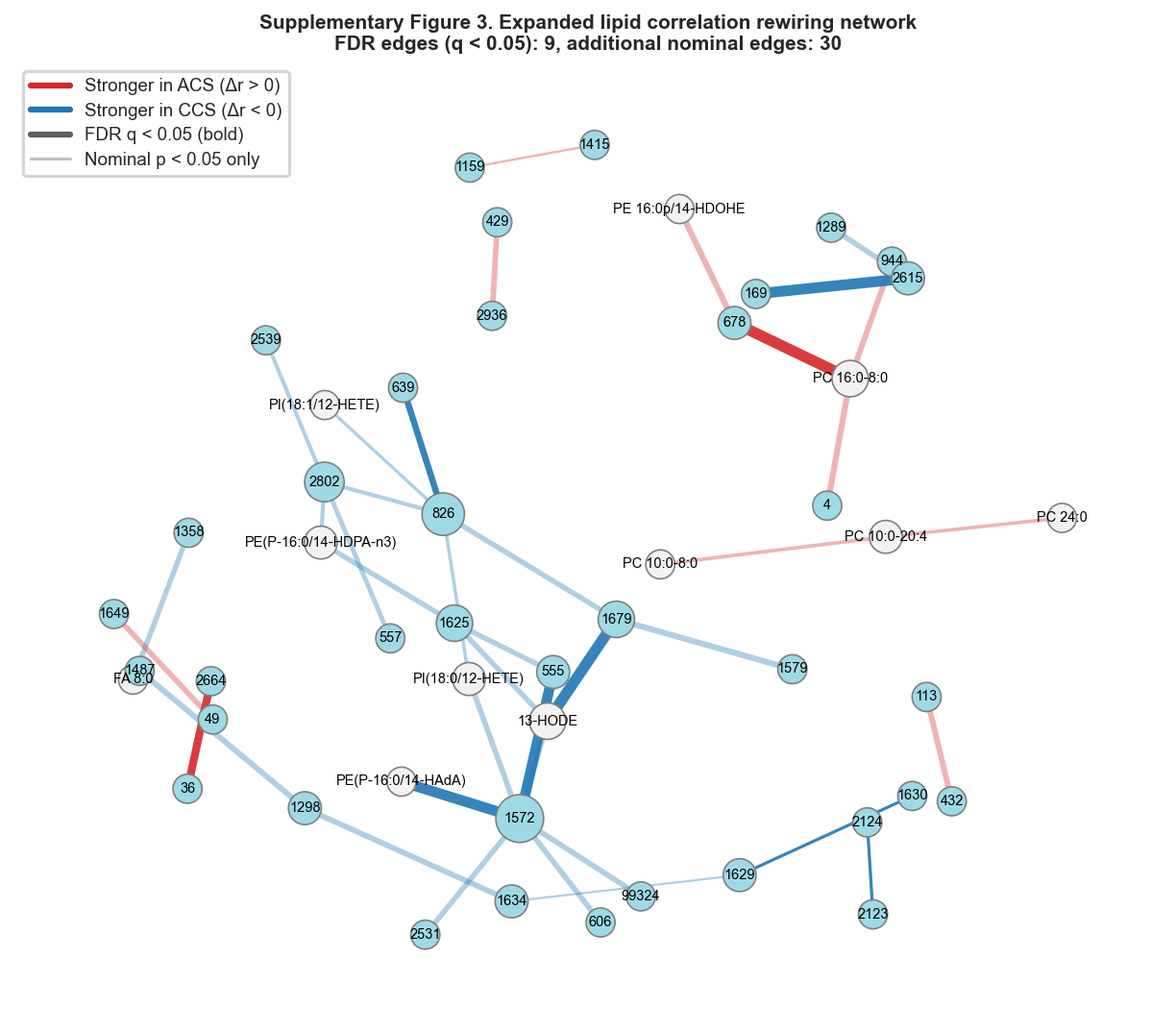

Supplement: Supplementary file 1 [file cells-15-01190-s001.zip › Kanpa_2026_Supplemental_Figure S3.png]

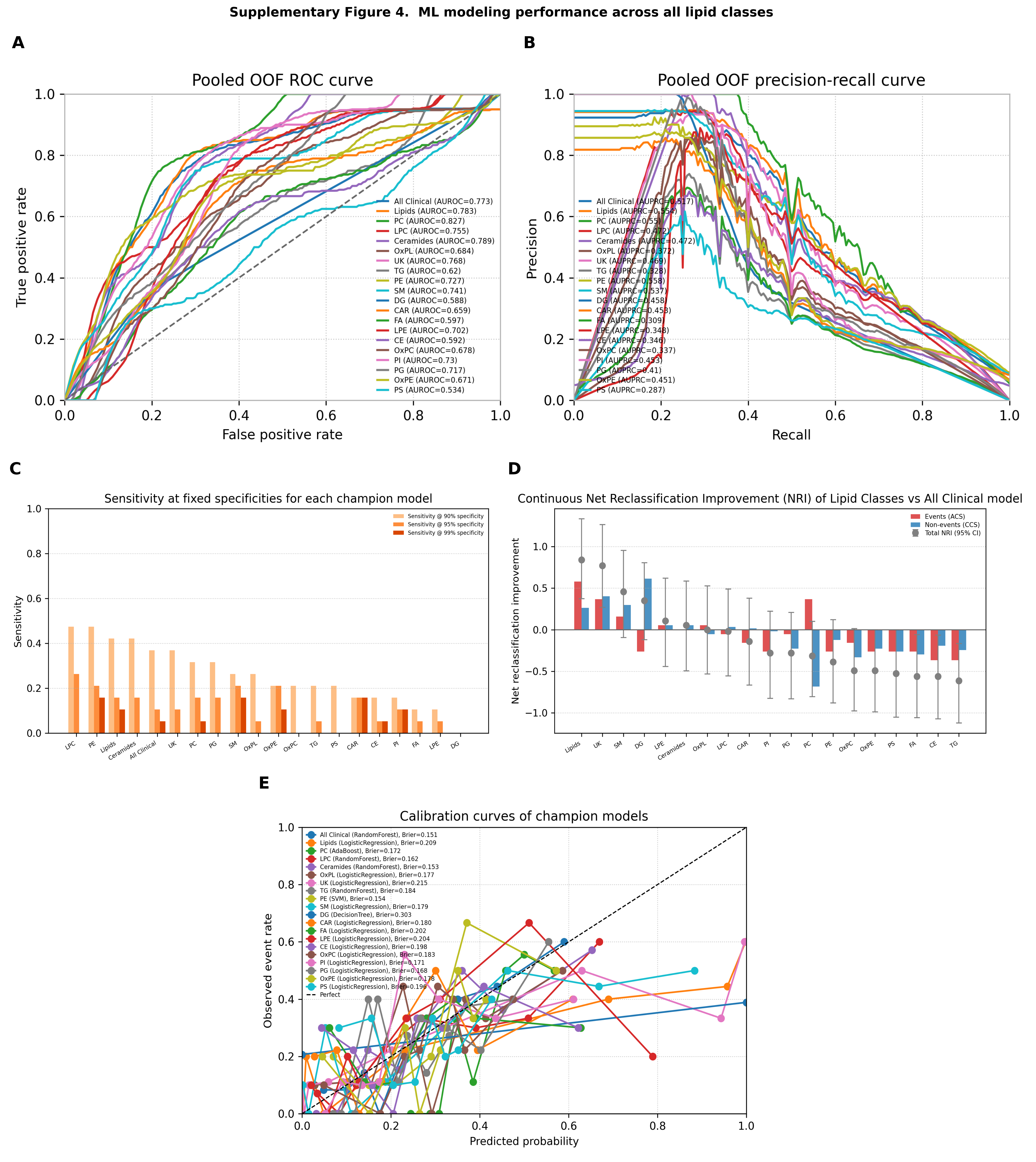

Supplement: Supplementary file 1 [file cells-15-01190-s001.zip › Kanpa_2026_Supplemental_Figure S4.png]

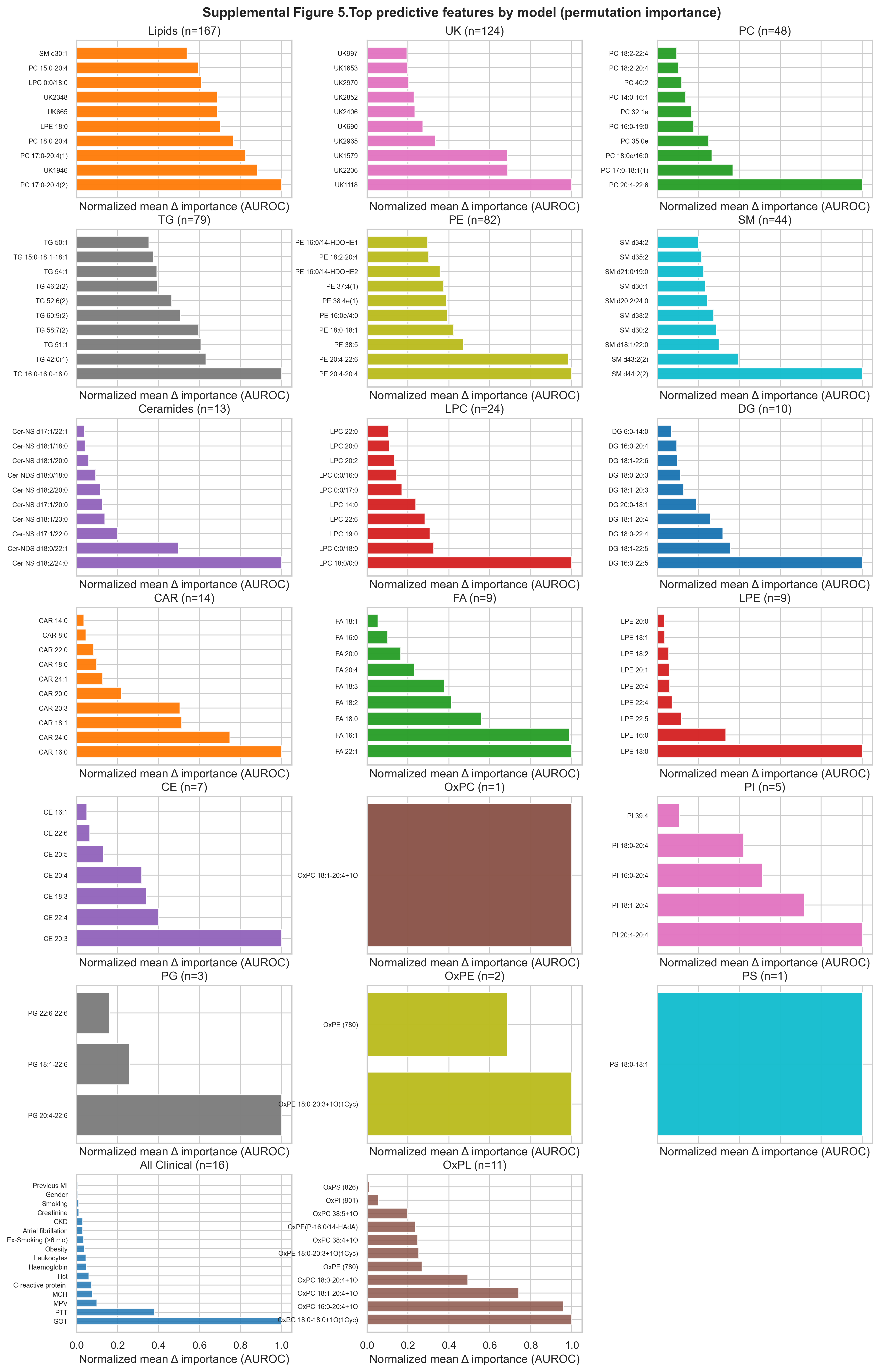

Supplement: Supplementary file 1 [file cells-15-01190-s001.zip › Kanpa_2026_Supplemental_Figure S5.png]
